# Efferent Pathways in Sodium Overload-Induced Renal Vasodilation in Rats

**DOI:** 10.1371/journal.pone.0109620

**Published:** 2014-10-03

**Authors:** Nathalia O. Amaral, Thiago S. de Oliveira, Lara M. Naves, Fernando P. Filgueira, Marcos L. Ferreira-Neto, Gerard H. M. Schoorlemmer, Carlos H. de Castro, André H. Freiria-Oliveira, Carlos H. Xavier, Diego B. Colugnati, Daniel A. Rosa, Graziela T. Blanch, Clayton L. Borges, Célia M. A. Soares, Angela A. S. Reis, Sergio L. Cravo, Gustavo R. Pedrino

**Affiliations:** 1 Center for Neuroscience and Cardiovascular Physiology, Department of Physiological Sciences, Biological Sciences Institute, Federal University of Goiás, Goiânia, GO, Brazil; 2 Faculty of Physical Education, Biological Sciences Institute, Universidade Federal de Uberlândia, Uberlândia, MG, Brazil; 3 Department of Physiology, Federal University of São Paulo, São Paulo, SP, Brazil; 4 Laboratory of Molecular Biology, Biological Sciences Institute, Federal University of Goiás, Goiânia, GO, Brazil; 5 Department of Biochemistry and Molecular Biology, Biological Sciences Institute, Federal University of Goiás, Goiânia, GO, Brazil; INSERM, France

## Abstract

Hypernatremia stimulates the secretion of oxytocin (OT), but the physiological role of OT remains unclear. The present study sought to determine the involvement of OT and renal nerves in the renal responses to an intravenous infusion of hypertonic saline. Male Wistar rats (280–350 g) were anesthetized with sodium thiopental (40 mg. kg^−1^, i.v.). A bladder cannula was implanted for collection of urine. Animals were also instrumented for measurement of mean arterial pressure (MAP) and renal blood flow (RBF). Renal vascular conductance (RVC) was calculated as the ratio of RBF by MAP. In anesthetized rats (n = 6), OT infusion (0.03 µg • kg^−1^, i.v.) induced renal vasodilation. Consistent with this result, e*x* *vivo* experiments demonstrated that OT caused renal artery relaxation. Blockade of OT receptors (OXTR) reduced these responses to OT, indicating a direct effect of this peptide on OXTR on this artery. Hypertonic saline (3 M NaCl, 1.8 ml • kg^−1^ b.wt., i.v.) was infused over 60 s. In sham rats (n = 6), hypertonic saline induced renal vasodilation. The OXTR antagonist (AT; atosiban, 40 µg • kg^−1^ • h^−1^, i.v.; n = 7) and renal denervation (RX) reduced the renal vasodilation induced by hypernatremia. The combination of atosiban and renal denervation (RX+AT; n = 7) completely abolished the renal vasodilation induced by sodium overload. Intact rats excreted 51% of the injected sodium within 90 min. Natriuresis was slightly blunted by atosiban and renal denervation (42% and 39% of load, respectively), whereas atosiban with renal denervation reduced sodium excretion to 16% of the load. These results suggest that OT and renal nerves are involved in renal vasodilation and natriuresis induced by acute plasma hypernatremia.

## Introduction

Hypernatremia constitute a challenge that threatens the survival of the organism. Despite the wide variation in daily intakes and losses of sodium, its concentration in the body fluids must be maintained within narrow limits. Therefore, maintenance of the plasma sodium concentration within a strict range is a central goal of homeostatic mechanisms [1], [2]. It is no surprise that multiple mechanisms are involved in the regulation of sodium concentration. This wide variety of mechanism ranges from localized and highly specific renal control of sodium loss to the complex regulation of ingestive behaviors [3]–[5].

The central nervous system (CNS) detects variations in the volume, tonicity, and composition of the extracellular compartment through peripheral and central receptors. Once detected, changes in plasma sodium concentration trigger centrally driven behavioral and neurovegetative adjustments in order to correct deviations [3], [4]. Behavioral responses consist of thirst and sodium appetite. The neurovegetative adjustments include changes in renal excretion of water and sodium. Indeed, hypertonicity reduces sympathetic renal nerve activity [5]–[8], induces renal vasodilatation [9]–[12], and increases release of vasopressin (VP), atrial natriuretic peptide (ANP) [13], [14] and oxytocin (OT) [15]–[18]. Together, these adjustments lead to natriuresis [19]–[22].

Renal vascular tone is a significant factor in the regulation of water and sodium excretion, and it is one of the variables adjusted in response to acute variations in the osmolality of the extracellular fluid [10], [11], [23]. The mechanisms involved in the genesis and maintenance of the renal vasodilation induced by increases in the plasma sodium concentration are complex and appear to involve neural and humoral mechanisms.

Oxytocin is well known for its role in uterine contraction [24] and milk ejection [25], but has several additional functions, many related with social behavior, maternal care, sexual behavior, learning and memory (for review see [26], [27]). In addition, OT seems to be involved in the responses to changes in body fluid osmolality. OT secretion is increased in response to alterations in the plasma sodium concentration [17], [28], and its infusion increases urinary volume [29], [30], glomerular filtration [29], [31], and urinary excretion of sodium in rats [30], [32]–[35]. These effects are mediated by the interaction of OT with the OXTR receptor, a G protein-coupled receptor that is coupled to phospholipase C through Gαq11 [26], [36].

Stimulation of the renal nerve reduces sodium excretion through secretion of renin, renal vasoconstriction, and increased tubular reabsorption of sodium [37]. Renal sympathetic activity is reduced by plasma hypertonicity, leading to renal vasodilation and natriuresis [5]–[8], [23]. However, to the best of our knowledge, there are no evidences that have evaluated if the secretion of OT is related with renal vasodilation and natriuresis induced by hypernatremia. Moreover, no other study sought to evaluate the interaction between renal sympathetic activity and secretion of OT on renal and cardiovascular responses induced by sodium overload.

Therefore, in the present study, we tested the hypothesis that OT and renal nerves may be involved in the cardiovascular and renal responses induced by acute hypernatremia. For this purpose, we assessed the effects of OXTR receptor blockade and renal denervation on renal vasodilatation and natriuresis induced by intravenous infusion of hypertonic saline. We also assessed the effects of OT on renal vascular tone and resistance.

## Results

### OXTR gene expression in the renal artery

**Figure 1:**
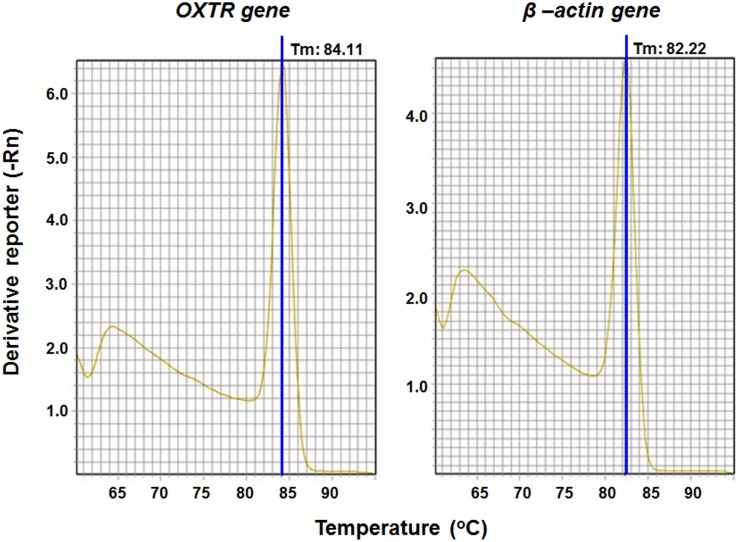
Melting curve showing the specific expression of the OXTR gene in renal arteries of rats after qRT-PCR analyses (A). Expression of the control gene B-actin was used (B).

The qRT-PCR analysis indicated that t OXTR was expressed in the renal artery (n = 10 arteries taken from 5 rats; Figure 1). These findings demonstrated that OXTR gene was expressed as β-actin, a constitutive gene. The Ct value was 32.01±2.1 for OXTR and 22.08±1.3 for β-actin. The Melting curve confirms the specific expression of OXTR gene in renal arteries (Figure 1A). As control, the expression of β-actin gene was used (Figure 1B).

### Effect of OT on renal vascular resistance and on renal artery rings

**Figure 2:**
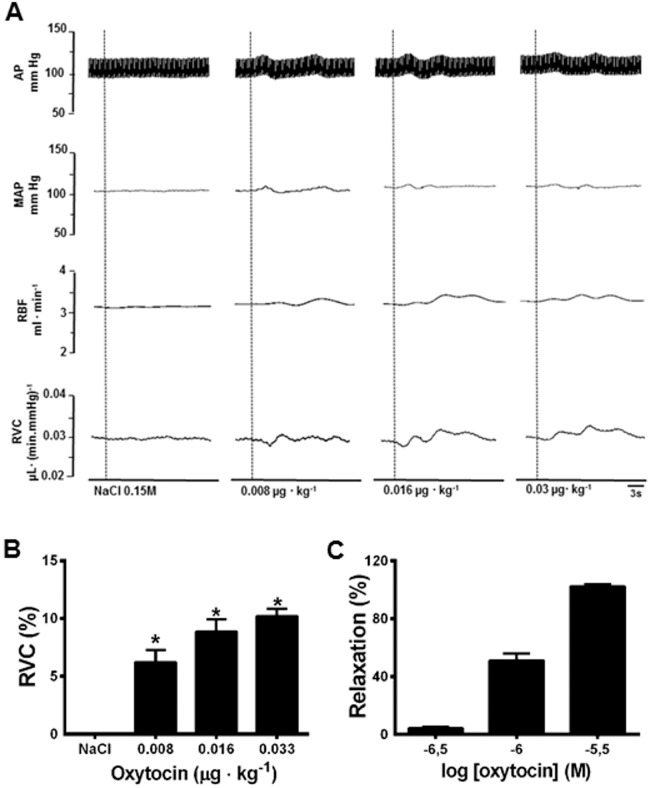
Typical tracing of changes in cardiovascular parameters induced by intravenous injection of vehicle (0.1 ml NaCl 150 mmol • l^−1^) and oxytocin (0.008, 0.016 e 0.03 µg • kg^−1^ b.wt., i.v.) in anesthetized rats (A). Effects of oxytocin or vehicle infusion on renal vascular conductance (RVC) in anesthetized rats (B; n = 6). Concentration–response curve for relaxation of the isolated renal artery by oxytocin (0.3×10^−6^ to 3×10^−6^ M; C - n = 6 rings taken from 5 rats). Error bars indicate S.E.M. Dashed line indicates oxytocin injection. AP = arterial pressure, MAP = mean arterial pressure, RBF = renal blood flow, RVC = renal vascular conductance. *p<0.05 compared to vehicle.

*In vivo* experiments demonstrated that the intravenous infusion of OT (8, 16, or 30 ng • kg^−1^, i.v.) in anesthetized rats (n = 6) did not change mean arterial pressure (MAP; Figure 2A). All three doses of OT increased renal blood flow (RBF; Figure 2A) and renal vascular conductance (RVC; Figure 2A and B). Renal vasodilation started immediately after infusion and lasted no longer than 30 s. *Ex vivo* experiments showed that OT produced a concentration-dependent relaxation in renal artery rings (EC50 = 0.91±0.07 µmol • l^−1^. Figure 2C; n = 6 rings taken from 5 rats) precontracted with 1 µmol • l^−1^ phenylephrine, suggesting a direct effect of OT on the renal artery.

### Effect of blockade of OXTR and VP-receptors on renal vasodilatation and vasorelaxation induced by OT

**Figure 3:**
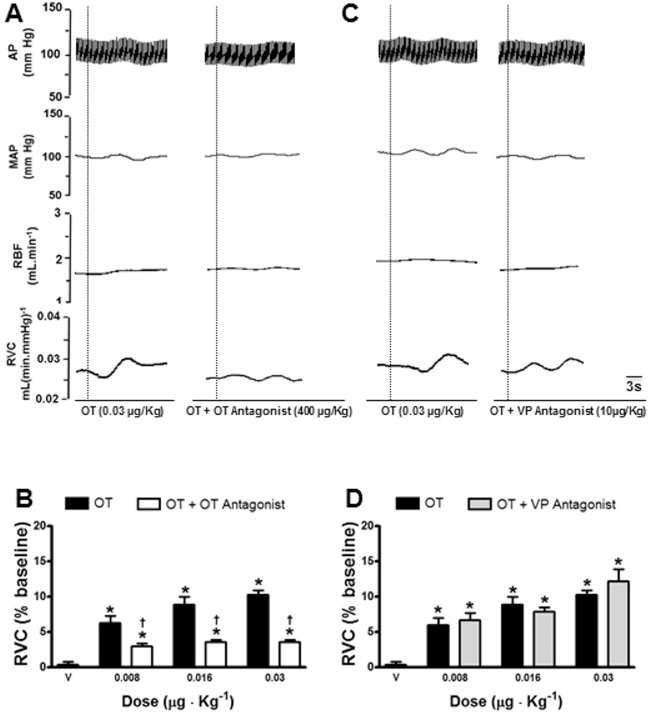
Typical tracing showing changes in cardiovascular parameters induced by intravenous infusion oxytocin (OT - 0.03 µg · kg^−1^; b.wt., i.v.; in 0.1 ml) before and after administration of OT-antagonist (Atosiban; 40 µg · kg^−1^ b.wt., i.v.; A) or VP-antagonist (Manning compound; 10 µg · kg^−1^ b.wt., i.v.; C). Effects of vehicle and OT infusion (0.008, 0.016 and 0.03 µg · kg^−1^ b.wt., i.v.) before and after administration of OT-antagonist (B) and VP-antagonist (D) on renal vascular conductance (RVC; n = 6). The results are expressed as the mean ± SEM. The dashed line indicates the OT infusion. AP = arterial pressure, MAP = mean arterial pressure, RBF = renal blood flow and RVC = renal vascular conductance. *different from vehicle (NaCl 0.15 M); †different from OT, both p<0.05.

**Figure 4:**
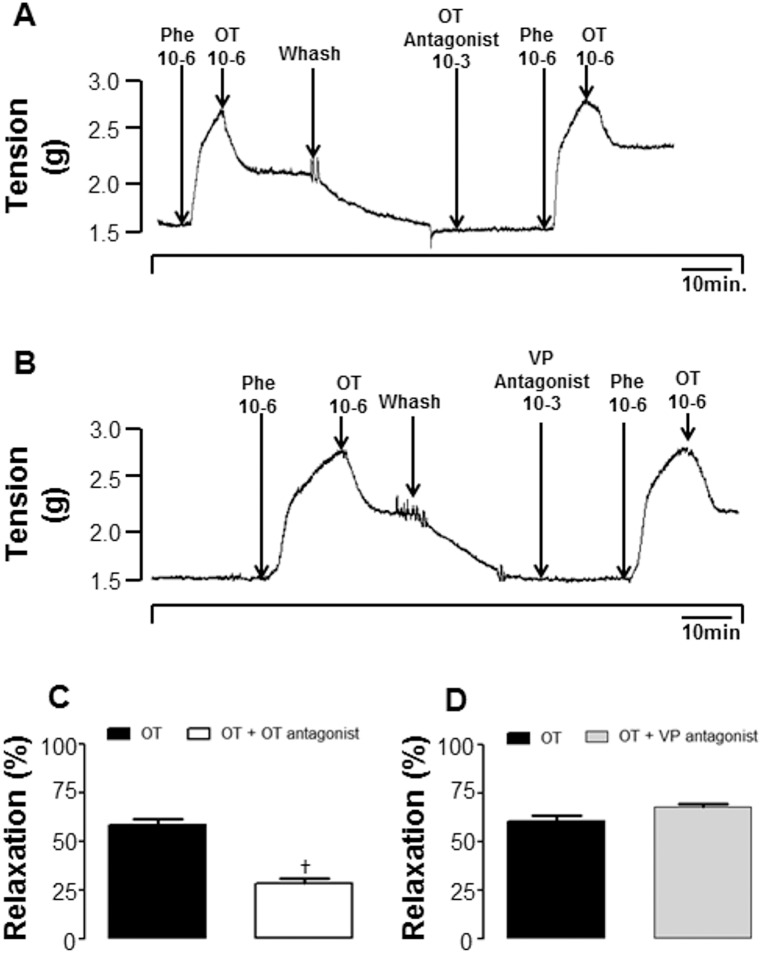
Typical tracing showing the effect of OXTR antagonist (A) and VP receptor antagonist (B) on vasorelaxation induced by OT (1 µM) in renal artery rings. Effects of pre-treatment with OT-Antagonist (C) and VP-antagonist on renal vasorelaxation induced by OT. †different from OT, p<0.05.

*In vivo* experiments demonstrate that the OXTR-antagonist, Atosiban, reduced OT-induced renal vasodilatation (Figure 3A and B, n = 6), whereas the V1 antagonist, Manning compound, did not affect renal vasodilatation induced by OT (Figure 3C and D, n = 6). *Ex vivo* experiments showed that the vasorelaxation induced by 1 µM OT was significantly reduced in the presence of the OXTR antagonist, Atosiban (1 mM, Figure 4A and C). The vasopressin V1 antagonist, Manning Compound (0.1 µM) did not reduce OT-induced vasorelaxation (Figure 4B and D), suggesting a direct effect of OT on OXTRs in the renal artery.

### Effects of hypertonic saline infusion on plasma sodium and blood hemoglobin concentrations

**Figure 5:**
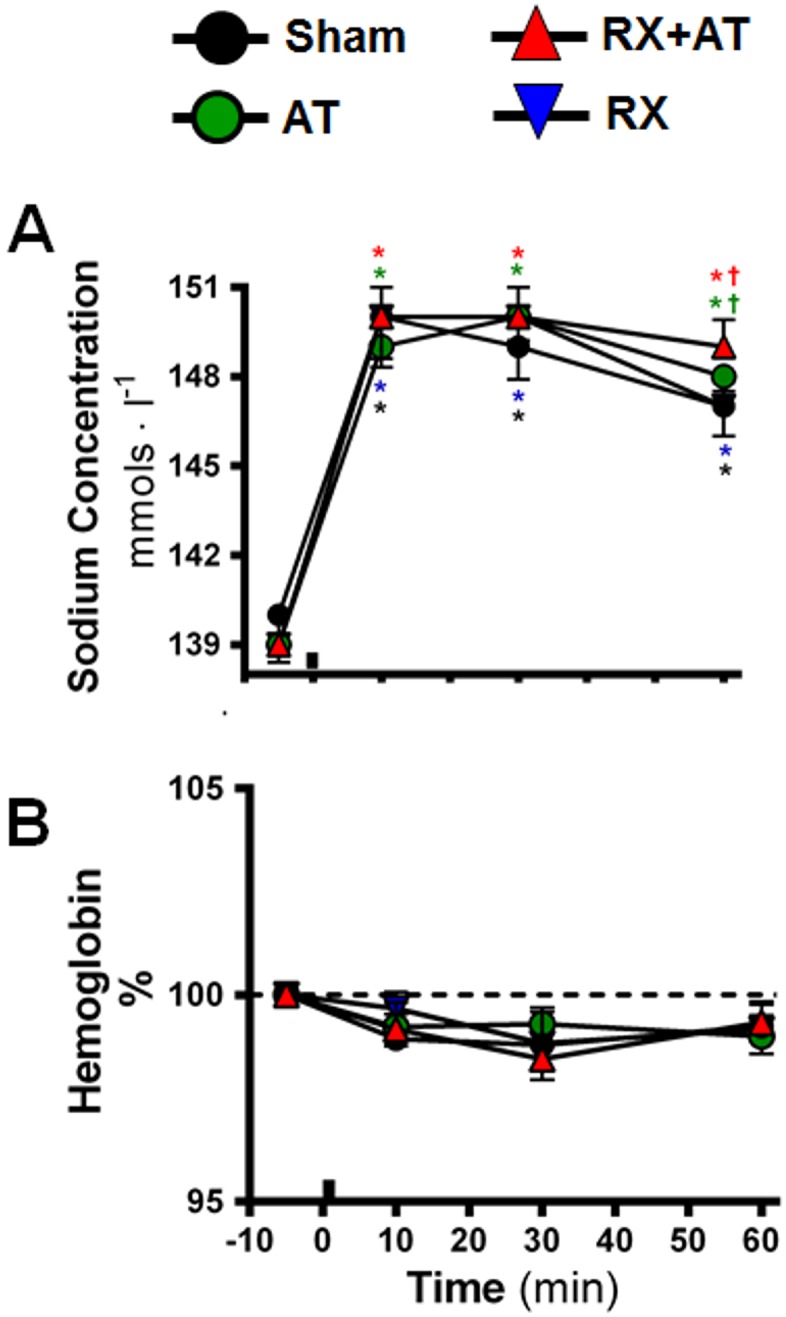
Effects of OXTR antagonist treatment (atosiban; AT), renal denervation (RX) and the combination of AT and RX (RX+AT) on changes in plasma sodium (A) and blood hemoglobin concentration (B) induced by HS infusion (3 M NaCl, 1.8 ml • kg^−1^ b.wt., i.v). Black bars indicate the moment of HS infusion. *different from baseline, †different from sham, both p<0.05.

The body weight and cardiovascular baseline values are shown in Table 1. These variables were similar in sham and rats subjected to OXTR-antagonist pretreatment (Atosiban; AT), renal denervation (RX) and renal denervation and atosiban pretreatment (RX+AT). In sham rats, intravenous infusion of hypertonic saline (HS) caused a sustained (>60 min) increase of plasma Na^+^ concentration (Figure 5A). In AT and RX+AT, plasma Na^+^ concentration remained significant higher than in sham rats (Figure 5A).

**Table 1:** Body weight and basal level of cardiovascular and renal variables in sham, rats pretreated with the oxytocin antagonist atosiban (AT), renal denervated rats (RX) and rats pretreated with atosiban and renal denervation (RX+AT).

Group	n	b.wt.	MAP	HR	RBF	RVC
		g	mm Hg	bpm	ml • min^−1^	mm Hg • (ml • min)^−1^
**Sham**	6	306±9	115±2	340±15	3.6±0.8	0.029±0.004
**AT**	7	307±6	113±5	367±12	4.5±0.7	0.038±0.006
**RX**	6	300±6	115±3	359±10	3.5±0.7	0.031±0.007
**RX-AT**	7	305±7	110±7	354±10	3.0±0.9	0.021±0.003

Values are means ± S.E.M. b.wt. = body weight, MAP = mean arterial pressure, HR = heart rate, RBF = renal blood flow, RVC = renal vascular conductance.

In sham rats, HS infusion did not change blood hemoglobin concentration (Figure 5B). Combining HS with AT, RX, or both did not alter changes in blood hemoglobin (Figure 5B).

### Effects of OXTR blockade and renal denervation on cardiovascular responses induced by HS infusion

**Figure 6:**
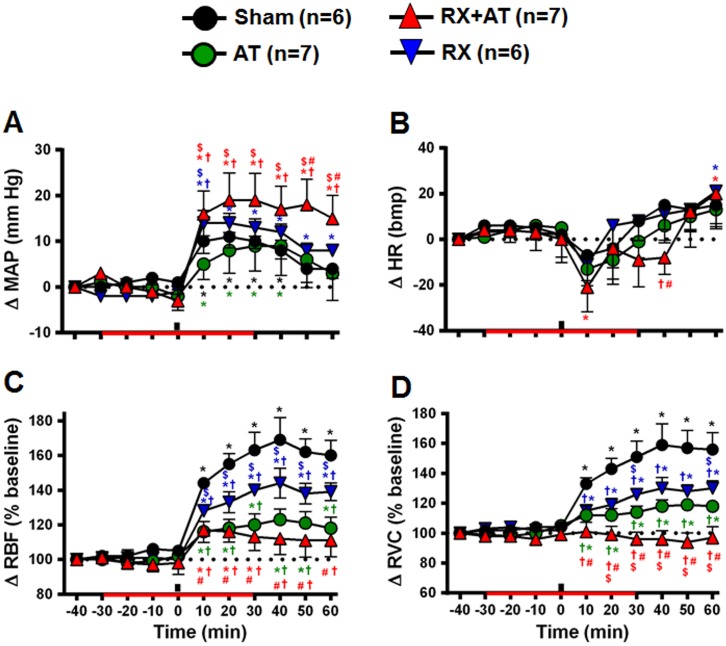
Effects of OXTR antagonist treatment (atosiban; AT), renal denervation (RX) and the combination of AT and RX (RX+AT) on cardiovascular changes induced by intravenous HS infusion (3 M NaCl, 1.8 ml • kg^−1^ b.wt., i.v). A) Mean arterial pressure (MAP). B) Heart rate (HR). C) Renal blood flow (RBF). D) Renal vascular conductance (RVC). Black bars indicate the moment of HS infusion. Red bars indicate the period of OXTR-antagonist infusion or saline (150 mmol · l^−1^ NaCl; Sham and RX groups). *different from baseline, †different from sham, $different from atosiban, #different from RX, both at p<0.05.

In sham rats (n = 6), HS infusion caused a mild increase in MAP (Figure 6A), without changing heart rate (HR; Figure 6B). Ten minutes after the HS infusion, RBF and RVC were increased, and they remained high after 60 min (Figure 6C–D). In AT rats (n = 7), changes in MAP and HR were similar to those observed in sham rats (Figures 6A–B), but the increases in RBF and RVC were much reduced (Figures 6C–D). In RX rats (n = 6), HS infusion did not change HR, but the pressor response (10 min after HS) was increased, and renal vasodilation was blunted (Figure 6). The combination of atosiban and renal denervation (RX+AT rats, n = 7) potentiated the hypertensive response (Figure 6A) and abolished the renal vasodilation induced by HS infusion (Figure 6C–D).

### Effects of blockade of OXTR and renal denervation on urinary excretion induced by HS infusion

**Figure 7:**
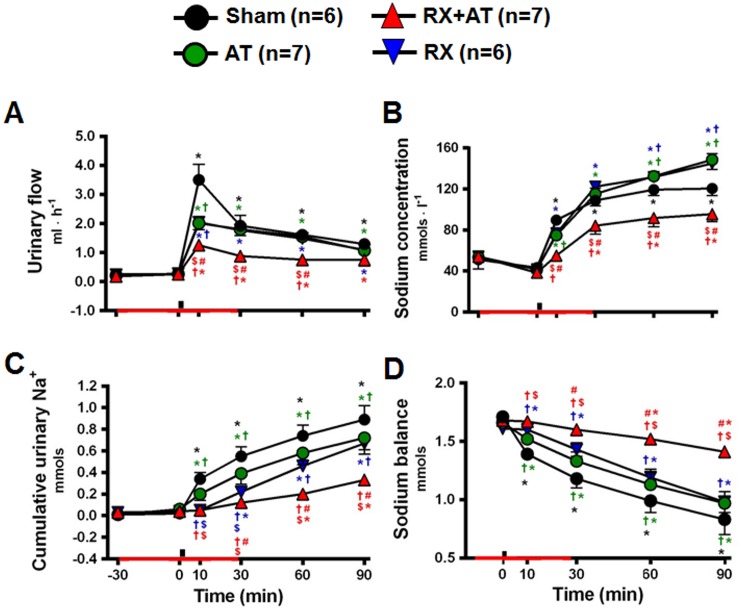
Effects of OXTR antagonist treatment (atosiban; AT), renal denervation (RX) and the combination of AT and RX (RX+AT) on urinary changes induced by intravenous HS infusion (3 M NaCl, 1.8 ml • kg^−1^ b.wt., i.v.). A) Urine flow. B) Urinary sodium concentration. C) Cumulative urinary sodium. D) Sodium balance. Black bars indicate the moment of HS infusion. Red bars indicate the period of OXTR-antagonist infusion or saline (150 mmol · l^−1^ NaCl; Sham and RX groups). *different from baseline, †different from sham, $ different from atosiban, #different from RX, both at p<0.05.

HS infusion increased urinary flow in sham rats (n = 6) at 10 min (Figure 7A). The diuresis caused by HS was reduced by Atosiban (n = 7) and by renal denervation (n = 6), and was reduced further by the combination of Atosiban and renal denervation (n = 7; Figure 7A). Urinary sodium concentration increased after infusion of HS. The increase of urinary sodium concentration after sodium overload was higher in AT and RX (Figure 7B), but it was reduced by the combination of RX+AT (Figure 7B). AT and RX slightly reduced the amount of sodium, and RX+AT reduced it further (Figure 7C). As a consequence, sham rats excreted 51% of the injected sodium within 90 min, AT rats excreted 42%, RX rats excreted 42%, and At+RX rats excreted only 16% of the load (Figure 7D).

### Effect of OXTR antagonist on cardiac mRNA for ANP

**Figure 8:**
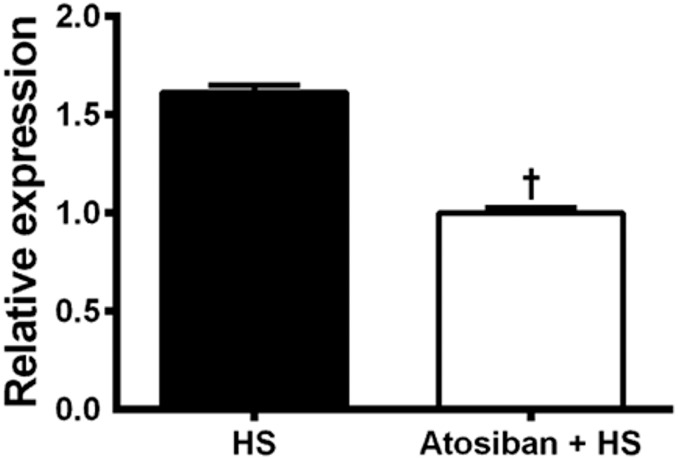
Effect of blockade of OXTR (Atosiban) on atrial natriuretic peptide (ANP) mRNA in the heart of rats submitted to HS infusion 3 M NaCl, 1.8 ml • kg^−1^ b.wt., i.v. ANP mRNA levels were assessed by the real-time quantitative reverse transcription polymerase chain reaction. The results are presented as mean ± standard deviation of the triplicates of independent experiments. †different from Sham, at p<0.05.

The effect of the OXTR antagonist, Atosiban, on cardiac expression of the ANP gene was measured by qRT-PCR. Figure 8 demonstrated that pre-treatment with OXTR antagonist reduced cardiac ANP gene expression in rats subjected to HS infusion.

## Discussion

Previous studies indicate that intravenous administration of OT increases urinary volume [29], [30], glomerular filtration [29], [31], and urinary excretion of sodium [17], [32], [34], [35]. However, the participation of this hormone in the regulatory mechanisms activated by an increase of plasma sodium concentration is yet to be fully clarified. The present study provides the following findings: i) the OXTR gene was expressed by the renal arteries; ii) administration of OT promoted renal artery relaxation *in vivo* and *ex vivo*; iii) blockade of OXTR, but not VP-receptors, reduced renal artery relaxation *in vivo* and *ex vivo* induced by OT; iv) blockade of OXTR and renal denervation reduced renal vasodilation and natriuresis induced by acute hypernatremia; v) the combination of OXTR antagonist and renal denervation completely abolished the renal vasodilation and further reduced natriuresis induced by sodium load; and vi) cardiac ANP gene expression induced by HS infusion was decreased in the presence of OT receptors antagonist. Collectively, these results suggest that the renal vasodilation and natriuresis induced by sodium overload is mediated by the combined action of OT and renal nerves.

Changes in the osmolarity of the extracellular compartment affect all tissues and may alter cell volume, metabolism, and function [1]. Acute alterations in osmolarity may induce convulsions, paralysis, coma, and in extreme conditions, death [2], [4]. Thus, the precise regulation of the osmolality and therefore intracellular volume is critical for survival. Disturbances in these regulatory mechanisms have been associated with the pathophysiology of cardiovascular diseases, particularly hypertension [38]–[40], and with diseases related to renal sodium retention, such as cirrhosis and heart failure.

The present study demonstrated that OT induced a concentration-dependent relaxation in renal arteries both *in vivo* and *in vitro.* The complex mechanisms involved in the genesis and maintenance of the renal vasodilation induced by increases plasma sodium concentration are poorly understood and seem to involve hormonal and autonomic mechanisms [8], [10], [14], [41], [42]. Our *in vitro* results show that OT can act on oxytocin receptors in the renal vasculature to promote vasodilatation. Experimental evidence has demonstrated that hypernatremia induces OT secretion [15], [17], [28]. Huang *et al*. (1995) [17] showed that infusion of hypertonic saline stimulates OT secretion and natriuresis. Our data showed that the blockade of OXTR abolished the vasodilation and reduced the natriuresis induced by a sodium overload. Together, these results indicate that OT is involved in the cardiovascular and renal responses to an increase in plasma sodium concentration. To the best of our knowledge, there is no other evidence that OT mediates renal vasodilation induced by hypernatremia. Although HS increased plasma sodium concentration, it did not significantly reduce blood hemoglobin, suggesting blood volume did not change. This is consistent with previous studies using the same protocol [10], [11], [13], [14], [23]. Therefore, it is likely that the cardiovascular and renal adjustments induced by HS infusion were due to increased sodium concentration rather than to volume expansion.

It has been proposed that the natriuresis and renal vasodilatation induced by OT are due to cardiac OXTR receptors that stimulate ANP release [30], [35], [43], [44]. In the present study we demonstrated that an OXTR antagonist reduced cardiac ANP gene expression induced by HS infusion. Our result is consistent with studies conducted by Haanwinckel *et al*. [30], who demonstrated that the intraperitoneal or intravenous administration of OT causes an increase in the plasma concentration of ANP. Moreover, Gutkowska and coworkers [44] demonstrated that OXTR were functional and important for the secretion of ANP, as OXTR blockade reduces the release of ANP induced by OT administration. Once in the circulation, ANP stimulates renal vasodilation and natriuresis [47]. We also demonstrated that OT can act directly on the renal arteries to cause renal relaxation. Overall, these findings suggest that OT can act both directly and indirectly (via ANP) to promote renal vasodilatation.

Neural mechanisms also regulate sodium excretion. Renal nerve stimulation promotes renin secretion, renal vasoconstriction, and tubular sodium reabsorption [37], reducing renal sodium losses. Plasma hypernatremia inhibits renal sympathetic activity, stimulating sodium excretion [5]–[7]. A previous study from our laboratory [8] has shown that renal denervation slightly reduces the renal vasodilation induced by sodium overload, suggesting this vasodilation is partly a consequence of renal sympathoinhibition. Furthermore, in the present study we demonstrated that a combination of renal denervation and OXTR blockade abolished renal vasodilation and sharply reduced the natriuresis. Taken together, these results suggest an additive effect of OT secretion and renal nerves on natriuresis and renal vasodilatation induced by a sodium overload. In the present study we also observed that pressor responses to a sodium overload are greater in rats subjected to renal denervation and blockade of OXTR. Several other studies that blocked hypernatremia-induced renal vasodilation similarly reported an increase in the magnitude and duration of the hypertensive response [7], [10]–[12]. A decade ago, it was proposed that hypernatremia initially stimulates secretion of vasoactive peptides (ANP and OT) and reduces renal sympathetic activity. This mechanism leads to reduction in renal vascular resistance and sodium reabsorption, which culminates in sodium excretion. If this initial mechanism fails to correct hypernatremia, a generalized increase in sympathetic activity increases blood pressure, leading to increases in renal perfusion pressure, glomerular filtration rate and consequently natriuresis [3]. In the light of these evidences, the increased pressor response to hypernatremia observed in animals subjected to renal denervation and blockade of OXTR could be due to the ineffectiveness of the initial mechanism, resulting in a generalized increase in sympathetic activity and consequent hypertension. It is noteworthy, however, that the results obtained in the present study also indicate that this pressure mechanism failed to stimulate sodium excretion, since only 16% of of sodium load was excreted during the next 90 min in rats subjected both renal denervation and blockade of OXTR.

Our results are compatible with the hypothesis that OT secretion and renal nerves play an important role in renal vasodilatation induced by hyperosmolarity. This strongly suggests that both humoral (oxytocinergic) and autonomic (sympathoinhibitory) mechanisms are important. The unbalance in one or both these mechanisms may contribute to several pathological conditions, including some models of experimental hypertension in rodents.

## Methods

### Animals and ethics statement

All experiments were conducted on adult male Wistar rats (280–350 g). Rats were obtained from the central animal house of the Federal University of Goiás. Experimental procedures were designed in strict adherence to the National Health Institute Guidelines for Care and Use of Laboratory Animals, and approved by the Research Ethics Committee of the Federal University of Goiás (protocol number 034/12).

### Molecular analysis of OXTR gene expression in the renal artery

Total RNA was extracted from ten renal artery using Trizol (Invitrogen Corporation, Carlsbad, CA, USA), chloroform was added (1∶5, v/v), and the aqueous phase was obtained after centrifugation (12,000×g, 15 min). RNA was precipitated with isopropanol for 15 min at room temperature, followed by centrifugation at 12,000×g for 10 min. The pellets were resuspended in water treated with 0.1% DEPC, and the RNA content was quantified by spectrophotometry.

For reverse transcription, 2 µg of total RNA was used as a template to synthesize cDNA. RNA was incubated with 1 µl oligo(dt) 15–20 (0.5 pmol, Fermentas - Thermo Scientific, Vilnius, Lithuania), and DEPC-water to a final volume of 12 µl, for 5 min at 65°C. The following reagents were then added to reach a final volume of 20 µl: 4 µl RT buffer (50 mM Tris–HCl, pH 8.3, 75 mM KCl, 3 mM MgCl_2_), 2 µl 10 mM dNTPs mix, 1 µl RiboLock RNase inhibitor (20 U/µl) and 1 µl RevertAid H Minus M-MuLV reverse Transcripase (200 µl/µl, Fermentas - Thermo Scientific, Vilnius, Lithuania). The mixture was incubated for 5 min at 25°C followed by 60-min at 45°C and cDNA synthesis was performeded at 70°C for 5 min.

qRT-PCR widely used for analysis of gene expression in tissue (Applied Biosystems Step One Plus PCR System, Foster City, CA, USA). We standardized a qRT-PCR method to analyze the expression of the OXTR, using β-actin as a constitutive gene. For OXTR we used as a forward primer: 5-CGA TTG CTG GGC GGT CTT CA-3 and as a reverse primer 5-CCG CCG CTG CCG TCT TGA G-3, resulting in a PCR product of 161 bp. For β -actin, we used as a forward primer 5-AGA GGG AAA TCG TGC GTG AC-3 and as a reverse primer 5-CAA TAG TGA TGA CCT GGC CGT-3, resulting in PCR product of 138 bp.

For qRT-PCR amplification, 2 µl of the cDNA pool resulting from the above reaction was used in a reaction containing PCR buffer Maxima SYBR Green/ROX qRT-PCR Master Mix (2X) (Fermentas – Thermo Scientific, Vilnius, Lithuania), 0.15 µM of each primer and water to produce a final volume of 25 µl. General qRT-PCR conditions were initial denaturation at 95°C for 10 minutes, followed by 40 cycles of 95°C for 15 s, annealing at 60°C for 1 min and extension at 72°C for 20 s. Amplification was completed with an additional step at 72°C for 5 min. A melting curve analysis was performed subsequent to amplification of the target sequence. The program for melting curve analysis was 95°C for 5 s, 65°C for 20 s, with 20°C/s transition rate and then ramping to 98°C at 0.2°C/s.

### Effect of OT on renal vascular resistance

Rats were anesthetized with 2–3% halothane in 100% O_2_ and catheters were inserted into the right femoral vein and artery. A catheter was inserted in the right jugular vein and advanced to bring the tip close to the right atrium. Halothane was replaced by sodium thiopental (40 mg • kg^−1^, i.v.; Cristália Ltda, Itapira, SP, Brazil). The trachea was cannulated to reduce airway resistance. Miniature ultrasonic transit-time flow probes (Transonic Systems Inc., Ithaca, NY, USA) were placed around the left renal artery. The body temperature was kept at 37±0.5°C with a thermostatically controlled heated table.

Arterial pressure was measured from the arterial cannula with a disposable pressure transducer. RBF was measured with an ultrasonic transit-time flowmeter (Transonic Systems, Inc., Ithaca, NY, USA). Data were recorded continuously with an MP150 analog-to-digital converter (Biopac Systems, Inc., Goleta, CA, USA). MAP and HR were determined from the pulsatile signal with AcqKnowledge software (version 3.7.1.; Biopac Systems, Inc., Goleta, CA, USA). The change of RVC was calculated as the ratio of RBF by MAP, and expressed as a percentage of baseline values.

Increasing doses of OT (0.008, 0.016 and 0.03 µg • kg^−1^, i.v.) were infused. In additional rats, the vasopressin V1 antagonist, Manning compound (10 µg • kg^−1^ b.wt.; in 0.1 mL) was injected i.v., followed by increasing doses of OT (0.008, 0.016 and 0.030 µg • kg^−1^, i.v.). In additional rats, the OXTR antagonist, Atosiban, was infused for 1 h, starting 30 min before increasing doses of OT infusion. The total amount of Atosiban infused was 40 µg • kg^−1^ in a volume of 3 ml • kg^−1^.

### Effect of OT on renal artery rings

Rats were anesthetized with halotane (2% in O_2_ 100%) (Cristália Ltda, Itapira, SP, Brazil) and decapitated. The renal artery was cleaned of adhering fat and connective tissue, removed, and cut in 2 mm long rings. Rings were placed in a warmed (37°C) organ bath containing 10 ml of modified Krebs-Henseleit solution (in mmol • l^−1^: NaCl = 130, NaHCO_3_ = 14.9, KCl = 4.7, KH_2_PO_4_ = 1.18, MgSO_4_ = 1.17, CaCl_2_ = 1.6, glucose = 5.5; pH = 7.4, bubbled with 95% O2 and 5% CO2). Rings were mounted on stainless steel hooks connected to isometric transducers. Tension was recorded with a data acquisition system (AVS Projetos, São Carlos, SP, Brazil). Rings were kept for 60 min with a tension of 1.5 g. During this period, the Krebs-Henseleit solution was changed every 15 min. Phenylephrine (1 µmol • l^−1^) was added to contract the rings. After tension stabilized (usually within 15 min), increasing concentrations (0.3 to 3 µmol • l^−1^) of OT were added. In additional experiments, the vasorelaxation induced by 1 µM OT was measured in the absence or presence of the OXTR antagonist, Atosiban (1 mM), or the V1 antagonist, Manning compound (0.1 µM). At the end of the experiment, the effectiveness of the endothelium was evaluated by observing the response to 10 µmol • l^−1^ acetylcholine.

### Effect of OXTR antagonist and renal denervation on cardiovascular and renal responses induced by hypertonic saline

Additional rats were anesthetized and fitted with cannulas in the femoral vein and artery and a flow probe around the renal artery, as described above. The bladder was catheterized through a suprapubic incision for collection of urine. About 60 min later, hypertonic saline (3 M NaCl, 1.8 ml • kg^−1^ b.wt., i.v.) was infused over 60 s. In some rats, the selective OXTR antagonist, [Mpa1, D-Try(Et)2, Thr4, Orn8]-OT (atosiban, Ferring, Sweden), was infused for 1 h, starting 30 min before the infusion of hypertonic NaCl. The total amount infused was 40 µg • kg^−1^ in a volume of 3 ml • kg^−1^. This dose has been shown previously to block the natriuretic effects of OT but not to interfere with VP-receptors [33], [43].

In another group of rats (RX), the left and right kidney innervation was removed surgically as previously described [8], [45]–[50]. Briefly, the renal arteries were dissected under a surgical microscope, and the renal nerves running along them were carefully removed. The connective tissue of the renal artery and vein were stripped and the renal artery was coated with a solution of 10% phenol in absolute alcohol.

In another group of rats, the innervation of the left and right kidney was removed and the animals were submitted to atosiban infusion 60 minutes after the renal denervation (RX+AT). In sham rats, the renal artery was dissected and renal nerves bundles were visualized and kept intact. These rats received the same volume (3 ml • kg^−1^) of saline (NaCl 150 mmols • l^−1^).

Blood samples (0.20 ml each) were withdrawn from the arterial cannula 10 min before and 10, 30, 60 and 90 min after i.v. infusion of hypertonic saline. The sample was replaced by an equal volume of sterile 150 mM NaCl. Blood hemoglobin concentration was measured immediately with a kit from Sigma (Drabkin’s reagent, kit 525). The rest of the sample was centrifuged for 5 min at 6000 g. The plasma was removed and stored at −20°C. Sodium concentrations in urine and plasma were measured with a flame photometer (model DM6, Digimed, São Paulo, Brazil).

The amount of sodium in the urine was determined by the product of urine volume and sodium concentration. Sodium balance was calculated as the difference between the amount infused and the amount excreted.

### Molecular analysis for ANP in heart

Rats were euthanized, the cardiac muscle was cut in small pieces and frozen in liquid nitrogen. The tissues were disrupted by vigorous mixing with glass beads as described before. qRT-PCR analysis was performed in triplicate from a cDNA pool of each treatment. For natriuretic peptide precursor A (ANP), we used as a forward primer 5-GGA TTT CAA GAA CCT GCT AGA-3, and as a reverse primer 5-GCT TCA TCG GTC TGC TCG C-3, resulting in a PCR product of 96 bp. For GAPDH, we used as a forward primer 5-GTC GGT GTG AAC GGA TTT G-3, and as a reverse primer 5-CCG CCG CTG CCG TCT TGA G-3, with a PCR product of 150 bp. All primers were specific for *Rattus norvegicus*. The data were normalized using the constitutive gene GAPDH as the endogenous control, amplified in each set of qRT-PCR experiments. Data were expressed as mean ± standard deviation of the triplicates of independent experiments. Standard curves were generated by diluting the cDNA solution 1∶5. Relative expression levels of genes of interest were calculated using the standard curve method for relative quantification [51].

### Data analysis

Results are presented as means ± S.E.M. Cardiovascular baseline values and the effects of OT infusion and OXTR and VP-antagonist were analyzed by one-way ANOVA. When groups differed significantly, the Bonferroni post hoc test was used. The effects of HS infusion on renal and cardiovascular parameters were analyzed by two-way ANOVA. When groups differed significantly, the LSD-Fisher post hoc test was used. Effects of OT infusion and OXTR and VP-antagonist were analyzed by one-way ANOVA. When groups differed significantly, the Bonferroni post hoc test was used. The EC50 for the effect of OT on vascular relaxation was obtained by linear regression of vascular relaxation and the log of the OT concentration using GraphPad Prism statistical software version 6.03 for Windows (GraphPad Software, Inc., San Diego, CA, USA). Molecular data were compared by the T test. A value of P<0.05 was considered to denote a significant difference.
